# A prospective evaluation of vitamin B1 (thiamine) level in myeloproliferative neoplasms: clinical correlations and impact of *JAK2* inhibitor therapy

**DOI:** 10.1038/s41408-018-0167-3

**Published:** 2019-01-24

**Authors:** Naseema Gangat, Amy Phelps, Terra L. Lasho, Christy M. Finke, Rangit Vallapureddy, Curtis A. Hanson, Rhett P. Ketterling, Mrinal M. Patnaik, Animesh Pardanani, Ayalew Tefferi

**Affiliations:** 1 Division of Hematology, Department of Internal Medicine, Mayo Clinic, Rochester, MN USA; 2 Division of Hematopathology, Department of Laboratory Medicine, Mayo Clinic, Rochester, MN USA; 3 Division of Laboratory Genetics and Genomics, Department of Laboratory Medicine, Mayo Clinic, Rochester, MN USA

Vitamin B1 (Thiamine) deficiency might result in Wernicke’s encephalopathy (WE); the latter was reported in clinical studies with the *JAK2* inhibitor fedratinib resulting in disruption of clinical development, despite efficacy in patients with myelofibrosis (MF)^1^. Existing data regarding risk of WE and thiamine deficiency in patients with myeloproliferative neoplasms (MPN) is conflicting with one study suggesting an increased risk (MPN vs. non-MPN: 1.09 vs. 0.39/1000 person-year, HR = 2.19)^2^ while none were noted to be thiamine deficient in another study involving 92 MPN patients^3^. Therefore, the objectives of the current study were, (i) to provide an accurate estimation of the incidence of thiamine deficiency in patients with MPN, (ii) assess clinical correlations of thiamine level and (iii) determine the impact of *JAK2* inhibitor therapy on thiamine level in MPN.

After Institutional review board approval, patients referred to our center with a suspected diagnosis of MPN were prospectively enrolled. All clinical and laboratory variables including treatment details were collected at the time of referral. Liquid chromatography-tandem mass spectrometry analysis of thiamine diphosphate in whole blood was performed at the time of referral. Reference range for thiamine level in whole blood was 70–180 nmol/L. The JMP® Pro 13.0.0 software from SAS Institute, Cary, NC, USA, was used for all statistical analysis.

A total of 115 patients were enrolled which included 28 patients without MPN and 87 with MPN. 87 MPN patients (median age, 65 years, 52% males) included 32 with primary myelofibrosis (PMF), 17 with polycythemia vera (PV), 17 with essential thrombocythemia (ET), 11 with post PV MF, 8 with post ET MF, and 2 with MPN-U. Details of patient characteristics including treatment details are summarized in Supplementary Table 1. The MPN and non-MPN patients were similar in their gender distribution, pattern of alcohol use, malnutrition and multivitamin use (*p* = 0.24, 0.12, 0.54 and 0.78 respectively), although the former were older (*p* = 0.09).

Median thiamine level of the study patients was 167 nmol/L (range; 60–442 nmol/L) with only two (1.7%) female patients displaying level below the normal reference range. One belonged to the non-MPN group (thiamine level 68 nmol/l); a 26 year old female with erythroid leukemia in the absence of alcohol use or malnutrition; the second patient (thiamine level 60 nmol/l) was a 38 year old female with ET, *CALR* type 2 mutated on hydroxyurea, without a history of alcohol use or malnutrition. Overall, both MPN and non-MPN patients displayed similar thiamine level (*p* = 0.89) (Fig. 1a).

**Fig. 1:**
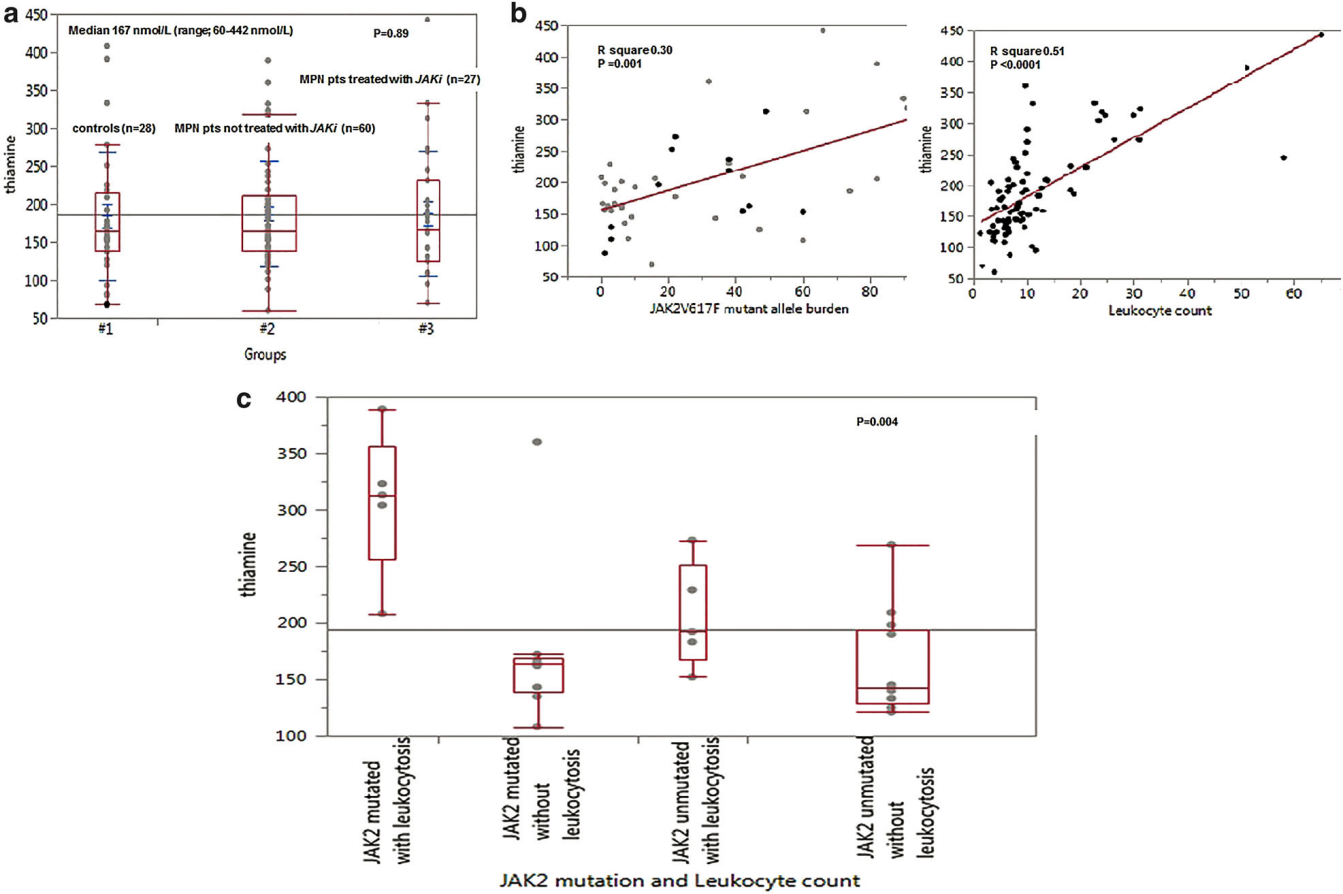
Thiamine levels in myeloproliferative neoplasms. **a** Comparison of thiamine level between patients with or without myeloproliferative neoplasm (MPN). **b** Correlation between thiamine level and (i) *JAK2V617F* mutant allele burden and (ii) leukocyte count. **c** Comparison of thiamine level in 32 patients with primary myelofibrosis stratified by *JAK2V617F* mutational status with or without leukocytosis > 10 × 10^9^/L

Amongst MPN patients, median thiamine level was similar amongst post ET MF, ET, PMF, PV, and post PV PMF patients (127, 162, 166, 190 and 209 nmol/L respectively, *p* = 0.13). No significant effect on thiamine level was apparent for gender, hemoglobin, platelet count, dynamic international prognostic scoring system score, unfavorable cytogenetics, palpable splenomegaly, transfusion dependence or constitutional symptoms (Supplementary Table 1). Interestingly, thiamine level was significantly higher in *JAK2* mutated cases (median 177 nmol/L vs 145 nmol/L in unmutated cases, *p* = 0.03). Furthermore, when analysis was restricted to PMF patients, thiamine level positively correlated with *JAK2V617F* mutant allele burden (*p* = 0.01) and leukocyte count > 10 × 10^9^/L (*p* = 0.03) (Fig. 1b). *JAK2* mutated PMF patients with leukocyte count > 10 × 10^9^/L depicted significantly higher thiamine levels (median 313 nmol/L) compared to *JAK2* mutated patients without leukocytosis (median 164 nmol/L) and *JAK2* unmutated patients with or without leukocytosis (median 192 and 143 nmol/L, respectively) (*p* = 0.004, Fig. 1c).

We further explored the impact of treatments particularly *JAK* inhibitor therapy on thiamine level in MPN patients. Treatment with hydroxyurea was documented in 35 patients with no significant impact on thiamine level (*p* = 0.28). 27 patients were on *JAK* inhibitors (median duration of exposure at referral; 41 months); 12 patients on ruxolitinib and 15 MF patients on momelotinib as part of a clinical trial. Thiamine level was found to be similar amongst *JAK* inhibitor treated vs untreated patients (median 166 vs 169.5 nmol/L respectively, *p* = 0.72). Additionally, annual thiamine measurements were performed on 15 patients treated with the *JAK* inhibitor momelotinib (median duration of therapy 52 months, range 18–60 months). Despite fluctuations in thiamine level, only two patients were noted to have thiamine level below normal (65 and 69 nmol/L at year 3), which subsequently improved to 101 and 121 nmol/L by year 4, while still on treatment (Fig. 2).

**Fig. 2:**
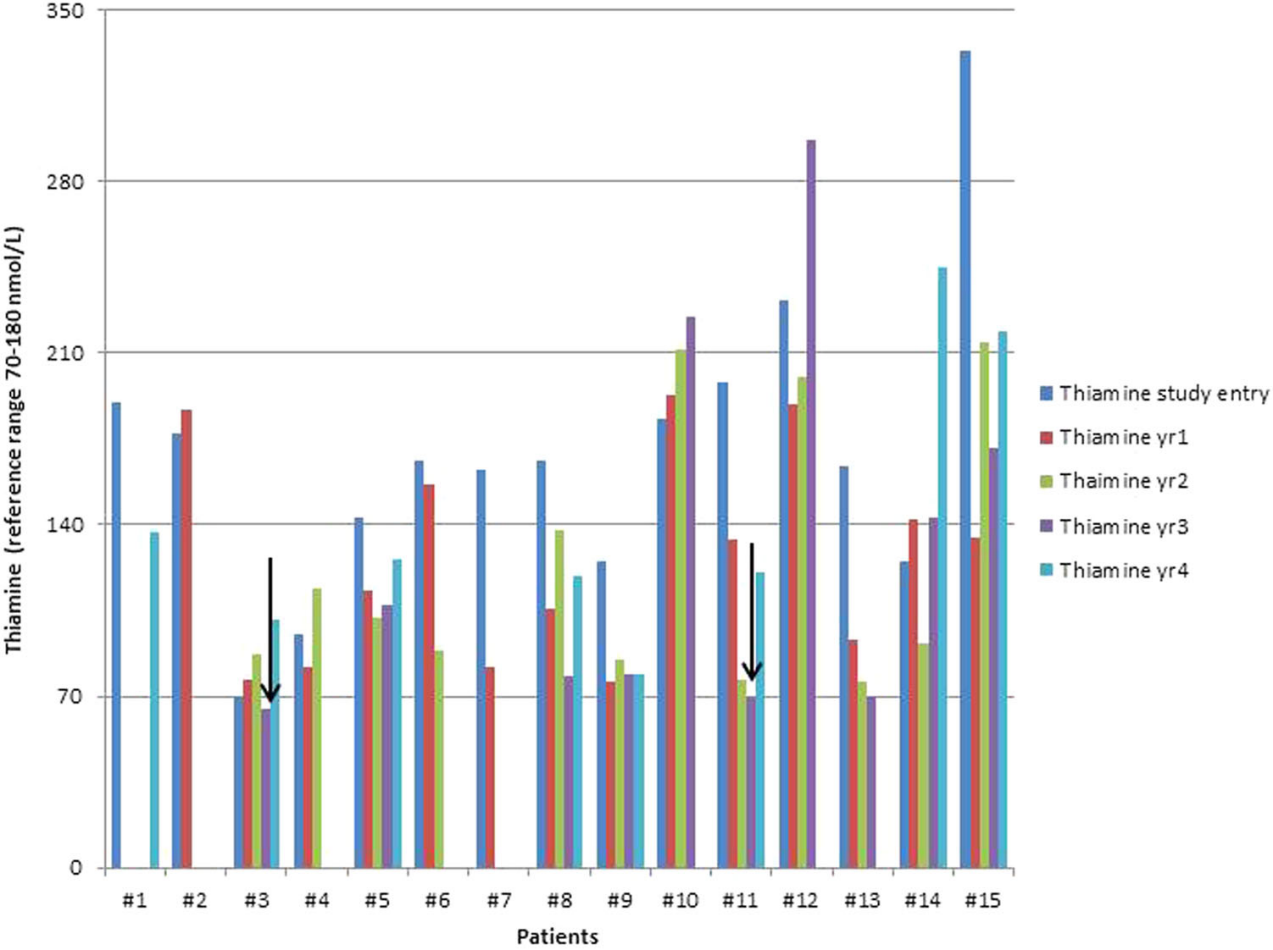
Thiamine level at study entry and annually in 15 patients with myelofibrosis treated with the *JAK2* inhibitor momelotinib

Thiamine plays a critical role as a co-enzyme for the activity of four key enzymes involved in cellular metabolism (pyruvate dehydrogenase, alpha-ketoglutarate dehydrogenase in the tricarboxylic acid cycle, transketolase within the pentose phosphate pathway and branched chain alpha-ketoacid dehydrogenase complex involved in amino acid catabolism). As a result it is pivotal in several functions within the central nervous system and immune systems^4^. Following intestinal absorption, it is transferred to blood mainly residing in erythrocytes (75%) and subsequently distributed to tissues via transporter proteins.

Herein, we corroborate findings of a prior study regarding the rarity of subnormal thiamine levels in MPN^3^. Interestingly, 46% of our patients demonstrated thiamine level above the reference range. These results are consistent with a study examining thiamine content in acute leukemia patients that found thiamine to be decreased in leukocytes and plasma but increased in erythroid cells^5^. Cancer cells frequently exploit thiamine dependent enzymes/pathways for proliferation which might explain higher thiamine levels noted in our patients^6^.

The correlation of thiamine level with both *JAKV617F* mutant allele burden and leukocytosis is intriguing in itself with a potential connection to the protective role of thiamine as an anti-inflammatory agent^7^. We clarify that the increased thiamine level noted in *JAK2V617F* mutant cases was independent of hemoglobin/hematocrit levels and transfusion needs. Whether the presence of the *JAK2V617F* mutation impacts intestinal uptake or distribution of thiamine to blood/tissues is unclear.

It remains contentious whether the *JAK2* inhibitor fedratinib is directly implicated in thiamine deficiency and subsequent development of WE. In a large series of 670 MPN patients treated on fedratinib clinical trials, only one of 7 suspected cases was confirmed to have WE^8^. Moreover, treatment with fedratinib did not decrease thiamine level. Likewise we found limited impact on thiamine level with long- term use of the *JAK2* inhibitor momelotinib.

In conclusion, thiamine deficiency is an infrequent occurrence in MPN regardless of treatment received. Our novel observation regarding the correlation of thiamine level with *JAK2V617F* mutant allele burden and leukocyte count requires further exploration.

## Supplementary information

Supp table 1
