# Supplementary material for: A prospective evaluation of vitamin B1 (thiamine) level in myeloproliferative neoplasms: clinical correlations and impact of JAK2 inhibitor therapy
Source: Blood Cancer J. 2019 Jan 24;9(2):11. doi: 10.1038/s41408-018-0167-3 (PMC6345855; doi:10.1038/s41408-018-0167-3)
Supplement: Supplementary file 1 — Supp table 1 [file 41408_2018_167_MOESM1_ESM.docx]

**Supplementary Table I. Clinical and laboratory characteristics including treatment details at referral for 115 patients with suspected myeloproliferative neoplasm**

|  | **Group #1**  Patients without MPN (control group)  (n=28) | **Group #2**  MPN patients not on *JAK* inhibitors  (n=60) | **Group #3**  MPN patients on *JAK* inhibitors  (n=27) | **Comparison of Groups #1,2 &3**  ***P*-value** | **Correlations with thiamine level in MPN patients**  (n=87)  ***P*-value** |
| --- | --- | --- | --- | --- | --- |
| Age in years, median (range) | 57.5 (26-81) | 63 (23-89) | 68 (38-88) | 0.07 | **0.035** |
| Gender, male, *n* (%) | 18 (64) | 31 (50) | 15 (55) | 0.24 | 0.32 |
| Thiamine level nmol/L, median (range) | 164.5 (68-408) | 164.5 (60-389) | 166 (70-442) | 0.93 | n/a |
| Malnutrition,  *n* (%) | 1(3.5) | 5(8) | 3(11) | 0.54 | 0.55 |
| Alcohol use, *n* (%) | 7(25) | 19(31) | 3(11) | 0.12 | 0.50 |
| Multivitamin, *n* (%) | 6 (21) | 17 (27) | 6(22) | 0.78 | **0.012** |
| MPN type, *n* (%)   - MPN-U - Prefibrotic MF - Post ET MF - Post PV MF - ET - PV - PMF | n/a | 2(2)  4(4)  8(9)  11(12)  17(19)  19(21)  28(31) | |  | 0.13 |
| Hemoglobin g/dl,  median (range) | n/a | 12.4 (6.8-19.7) | |  | 0.09 |
| Leukocyte count x10^9^/L,  median (range) | n/a | 8.1 (1.3-65) | |  | **<0.0001** |
| Platelet count x10^9^/L,  median (range) | n/a | 324 (34-1258) | |  | 0.19 |
| Lactate dehydrogenase (LDH), U/L  median (range)  N evaluable=77 | n/a | 367 (100-2409) | |  | 0.08 |
| Palpable splenomegaly, *n* (%) | n/a | 39(44) | |  | 0.19 |
| Constitutional symptoms, *n* (%) | n/a | 16(18) | |  | 0.11 |
| Transfusion dependent, *n* (%) |  | 6(7) | |  | 0.87 |
| DIPSS category,  N evaluable =32   - Low, *n* (%) - Intermediate-1, *n* (%) - Intermediate-2, *n* (%) - High, *n* (%) |  | 7(22)  14(44)  11(34)  0(0) | |  | 0.40 |
| Thrombosis at or after diagnosis, *n* (%)   - Arterial thrombosis - Venous thrombosis | n/a | 10(11)  5/10(50)  5/10 (50) | |  | 0.64 |
| Cytogenetics, abnormal  *n* (%)  N evaluable =66   - Unfavorable, *n* (%) - Very high risk (VHR) karyotype, *n* (%) | n/a | 25(38)  5/25(20)  4/25(16) | |  | 0.96  0.22 |
| Driver mutations   - *JAK2V617F* mutated, *n* (%) - *CALR* mutated*, n* (%) - *MPL* mutated, *n* (%) - Triple negative, *n* (%) - *JAK2 exon 12* mutated*, n* (%)   *JAK2V617F* mutant allele burden | n/a | 59(66)  12 (14)  9(10)  5(6)  1(1) | |  | **0.034**  **<0.0001** |
| Treatment, *n* (%)   - Aspirin - Hydroxyurea - Ruxolitinib - Momelotinib | n/a | 56(63)  58(65)  37(42)  12(13)  15(17) | |  | 0.85  0.28 (hydroxyurea)  0.72 (*JAKi*) |

MPN- myeloproliferative neoplasm, MPN-U- myeloproliferative neoplasm unclassified, MF- myelofibrosis, post ET MF- post essential thrombocythemia MF, post PV MF- post polycythemia vera MF, ET- essential thrombocythemia, PV, polycythemia vera, PMF- primary myelofibrosis, n/a- not applicable

Group#1 (control group): 18 patients with secondary erythrocytosis, 7 patients with a hematological malignancy other than MPN (2 patients with MDS/MPN overlap, 1 patient each with myelodysplastic syndrome, bone marrow failure, erythroid leukemia, mantle cell lymphoma, non-Hodgkin’s lymphoma), and 3 patients with no hematological disorder.

DIPSS: Dynamic international prognostic scoring system.

Unfavorable karyotype: sole abnormalities of +8 or 7q-, sole translocations not involving chromosome 1, sole abnormalities not otherwise classified, monosomal karyotype without VHR abnormality, complex non-monosomal without VHR abnormality, single/multiple 5q- abnormalities, two abnormalities without VHR abnormality.

Very high risk karyotype: single/multiple abnormalities of -7, inv(3)/3q21, i(17q), 12p-/12p11.2 or 11q-/11q23, single/multiple autosomal trisomies other than +9 and +8.
